# Teen Mental Health First Aid for years 7–9: a description of the program and an initial evaluation

**DOI:** 10.1186/s13033-019-0325-4

**Published:** 2019-11-16

**Authors:** Laura M. Hart, Kathy S. Bond, Amy J. Morgan, Alyssia Rossetto, Fairlie A. Cottrill, Claire M. Kelly, Anthony F. Jorm

**Affiliations:** 1 Population Mental Health Group, Centre for Mental Health, Melbourne School of Population and Global Health, University of Melbourne, Carlton, Australia; 2 School of Psychology and Public Health, La Trobe University, Bundoora, Australia; 3 Mental Health First Aid Australia, Parkville, Australia; 4 Mental Health First Aid England, London, UK; 5 School of Psychology, Deakin University, Geelong, Australia

**Keywords:** Mental Health First Aid, Mental health literacy, Stigma, Adolescents, Secondary school

## Abstract

**Background:**

A *teen Mental Health First Aid* training course for high school students in years 10–12 (tMHFA 10–12) has previously been evaluated in uncontrolled and randomized controlled trials and found to improve Mental Health First Aid intentions, mental health literacy and to reduce stigma. This 3 × 75-min course has more recently been adapted for younger students in years 7–9 (tMHFA 7–9). The present study reports an initial uncontrolled trial of this new training course which aimed to assess feasibility and acceptability of the course and test effects on knowledge, attitudes and behaviour.

**Methods:**

An uncontrolled trial was carried out in five schools with measures taken at pre-test, post-test and 3-month follow-up. The outcomes measured were: quality of first aid intentions to help peers, confidence in helping, stigmatising attitudes, recognition of anxiety disorder, number of adults thought to be helpful, help-seeking intentions, quality of support provided to a peer, quality of support received, and psychological distress. Questions were also asked about satisfaction with the course.

**Results:**

There were 475 students (mean age 13.86 years) who provided data at baseline, with 76% of these providing data at post-test and 75% at follow-up. Sustained changes at follow-up were found for: number of adults thought to be helpful, some components of stigma, recognition of anxiety disorder, and quality of support provided to a peer. However, there was an unexpected decline in willingness to tell others about a mental health problem. Most students found the information presented to be new, easy to understand, and useful.

**Conclusions:**

The tMHFA 7–9 training course produced some positive changes that were sustained over 3 months. However, the changes were not as strong as previously found for older high school students, suggesting the need for further refinement of the course.

## Background

Half of all lifetime mental illnesses emerge by the age of 14 and three quarters by the age of 24 [1]. As a key developmental period, the onset of mental health problems during adolescence can lead to significant and long-lasting impacts, including poorer mental health outcomes and adverse impacts on employment, education and social participation [2, 3]. Early intervention is critical to minimising the poor outcomes associated with adolescent mental illness [2], though many who meet diagnostic criteria do not receive appropriate treatment [4, 5]. Adolescents face a range of barriers to seeking professional help [6] including stigmatising attitudes, having a preference for self-reliance, and lacking knowledge about the signs and symptoms of mental health problems or when and how to seek professional help [6, 7].

When it comes to seeking help for mental health problems, adolescents are most likely to turn to their friends or family [8–10]. This help-seeking behaviour has implications for the type, quality and timeliness of support received, as adolescents often lack the skills and knowledge to provide appropriate assistance to a friend with mental health problem. For example, the quality of adolescents’ intended and actual support provided to peers has been found to be poor, particularly with regards to recommending professional help, which is often absent [11]. Peers failing to recommend that their friend get an adult involved to assist with their mental health is problematic because delayed intervention is associated with poorer treatment response, lower rates of remission, and a higher recurrence of illness [12, 13]. To be able to adequately support their peers, it is therefore critical that adolescents have good mental health literacy—the knowledge and beliefs about mental illness that aid recognition, management and treatment seeking [14].

School-based training programs for adolescents are recognised as an important strategy for improving mental health outcomes [15]. In 2010, a Delphi expert consensus study was undertaken to determine the key messages adolescents should be taught in a tailored mental health training program to increase support for peers with a mental health problem [16]. It was identified that there was need for separate courses for young adolescents (aged 12–15) and older adolescents (aged 16–18) due to differences in levels of maturity, social and cognitive capacity, and ability to take on particular help-seeking responsibilities [16]. Based on the results of this Delphi study, the teen Mental Health First Aid (tMHFA) program was created to teach high school students how to support a peer who may be developing a mental health problem or experiencing a mental health crisis. The program is comprised of two courses: one for older students aged 16–18 who are in years 10–12 in Australia (tMHFA 10–12) [18], and one for younger students aged 12–15 who are in years 7–9 in Australia (tMHFA 7–9) [17]. Both courses aim to teach adolescents how to: (a) recognise the signs that a peer may be developing a mental health problem, (b) talk to a peer about mental health and seeking help, (c) find appropriate resources about mental illness and professional help, and (d) respond in a crisis situation. The primary focus is on using an action plan to provide initial support to a peer until a responsible and trusted adult can become involved [16]. The tMHFA courses do not focus on specific mental illnesses; rather they teach students to recognise whether a friend may be exhibiting general signs of a mental health problem [17, 18]. In the tMHFA 7–9 course, crises are not emphasised, and more attention is paid to understanding the emergence of mental ill-health, in line with the expert consensus recommendations from the Delphi study [17, 18]. Courses are delivered by trained and accredited MHFA Instructors with expertise in adolescent mental health and experience in working with young people.

The efficacy and feasibility of the tMHFA 10–12 course has been evaluated in an uncontrolled trial and a cluster randomized controlled trial. In the uncontrolled trial, students with a mean age of 16 years were assessed at pre-test, post-test and 3-month follow-up [19]. The course was found to be acceptable to students, with the majority finding the course easy to understand, well presented and enjoyable. Improvements were found in mental health literacy, confidence in providing help, help-seeking intentions and student mental health, while stigmatizing attitudes reduced. The more recent randomised controlled trial assessed students at pre-test, post-test and 12-month follow-up. This trial found that the course significantly increased helpful Mental Health First Aid intentions, confidence to support a peer experiencing a mental health problem and number of adults rated as helpful, and decreased stigmatizing attitudes and harmful first aid intentions [20]. Many of these outcomes were also maintained at 12-month follow-up [21].

The aim of the current study was to conduct a pilot trial to test the feasibility and acceptability of the teen MHFA course for students in years 7–9, assess whether the course engages young people and contains appropriate and useful materials, and examine its impact on students’ knowledge, attitudes and behaviour.

## Methods

### Participants

Year 8 students (ages 12–15 years) were recruited from Australian schools in the state of Victoria. Schools were eligible to participate if they were willing to withhold other mental health training until the completion of the 3-month follow-up questionnaire and if they had not provided similar mental health literacy programs to year 8 students in the last 12 months. Five schools agreed to participate: four metropolitan government schools and one regional independent Catholic school. Details of the sociodemographic characteristics of the five schools are given in Additional file 1.

All students in the year 8 cohort at participating schools were offered the tMHFA 7–9 training program, whether or not they elected to participate in the evaluation research. The schools were also offered one free Youth MHFA course for parents and teaching or well-being staff involved with the year 8 students receiving training. Parents and school staff were also invited to complete a questionnaire with feedback on their perceptions of the students’ training. All intervention and questionnaire administration sessions were conducted between March and November 2017 (in Australia, school years run between February and December).

### Intervention

The tMHFA 7–9 training involved three 75-min classroom sessions delivered during normal school hours by an accredited MHFA Instructor with specific expertise in youth mental health. Classes consisted of 15–25 students and, in most cases, their regular classroom teacher was present during the training. Students who did not have parental consent to attend the training were given alternative activities. The manualised training involved a PowerPoint presentation, film clips, small group activities, group discussion and role-plays. Students received a booklet for use during sessions which they could keep for reference after the course [17]. Instructors were provided with training and a teaching manual to guide course delivery and ensure fidelity. Course content is outlined in Table 1.

**Table 1:** Content and structure of the tMHFA 7–9 training

Session 1	Session 2	Session 3
Topics presented		
Mental health Helpful and unhelpful thinking Mental health problems Appropriate help The importance of helping your friends	When should I do something? Teen MHFA Action Plan Helping someone who is suicidal	Putting what you’ve learned into practice Teen MHFA Action Plan Helping a friend with a mental health problem Looking after yourself
Films		
What it was like for me—Part 1 (lived experience film; 4:39)	Seeking help from a professional (filmed discussion with professionals 6:14) I’m fine (scripted drama film; 7:20)	What it was like for me—Part 2 (lived experience film; 4:34) What it was like for me—Part 3 (lived experience film; 5:35)
Session activities		
Unhelpful thinking How mental health problems affect young people	Review quiz Helpful people Looking for warning signs Helping James	Review quiz Helping Kasey Taking care of yourself

**Fig. 1:**
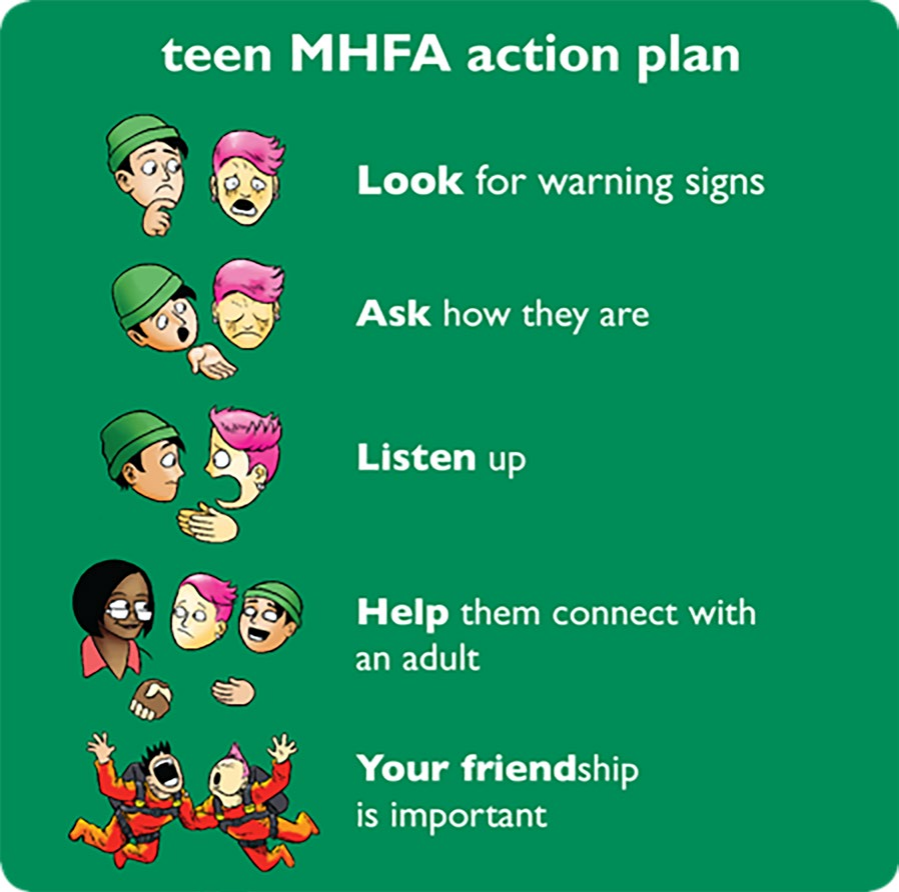
Teen Mental Health First Aid Action Plan. Students are taught a 5-point action plan which is shortened to the mnemonic ‘Look, Ask, Listen, Help Your Friend

The fundamental teaching of MHFA training is an action plan. The tMHFA 7–9 action plan is based on the key messages for adolescents providing Mental Health First Aid to a peer derived from a Delphi expert consensus study [16]. The tMHFA action plan consists of five first aid strategies and is taught in a mnemonic (see Fig. 1). The content and materials were initially piloted with two groups of year 7 students (n = 40), whose feedback on how to improve the training was incorporated. It was then delivered to five classes of year 8 students (n = 100) and the course was further refined before the uncontrolled evaluation began. A core message of tMHFA 7–9 training is to seek assistance from a responsible and trusted adult when a peer is experiencing a mental health problem. For this reason, the Youth MHFA course was also delivered to staff and parents at participating schools [22], to ensure that there were trained adults within each school who could be called upon to assist an adolescent by providing support and facilitating appropriate referral pathways.

### Procedure

Mental Health First Aid Australia maintains a database of individuals who are active and accredited in presenting the tMHFA 10–12 courses. An email was forwarded to all these instructors, calling for expressions of interest from schools that instructors had existing relationships with, to participate in the current study. Schools expressing interest in participating were then approached by the research team, and a meeting was held, usually with an assistant principal and lead well-being staff member. The research team described the tMHFA 7–9 course and the research, and answered any questions. If all parties were happy to proceed, a memorandum of understanding was signed by the research team and the school principal.

Passive, opt-out parental consent was used at the four government schools, while the independent Catholic school required opt-in consent. The research team therefore used multiple methods to ensure that the school community was aware of the upcoming training course and evaluation questionnaires. At least 3 weeks before the tMHFA 7–9 sessions were due to begin, a plain language statement, opt-out (government schools) or opt-in (Catholic school) consent forms were sent to the parents of each year 8 student, both electronically and in hard copy. Parent, teacher and student information sessions were also held. Where possible parent information sessions were held in combination with another school event (e.g. parent and teacher conferences) to increase the number of parents who attended the session. Any students who had a returned opt-out form or declined active parental consent were given alternative activities by school staff during questionnaire and training sessions.

Evaluation questionnaires were administered during regular class time. The research team were not given access to students’ contact details; instead a generic electronic link to the questionnaires was forwarded by the host school to student emails, or placed on the school intranet homepage for students to access. Questionnaires were administered online via the website SurveyMonkey.com, and completed by students either using a personal laptop or tablet, or a laboratory computer. Where students did not have access to an electronic device, they completed paper copies and data was later entered by research staff. Completion of each questionnaire took approximately 30 min. Students entered their own unique identifier (student ID) at the beginning of each questionnaire and this was used to track completion and match questionnaires over time.

Most students completed the baseline questionnaire immediately before participating in the first tMHFA 7–9 session. However, two schools had some students complete the questionnaire up to 6 weeks prior to the first session, due to timetabling constraints. For the majority of students, the three 75-min sessions of the intervention were held once per week for 3 weeks, though depending on timetabling constraints at each school, some sessions were run with a minimum of 3 days, and a maximum of 2 weeks, between each session.

For most students, the post-course questionnaire was administered immediately after the third and final tMHFA 7–9 session, but some students completed the questionnaire up to 1 week after the last session. The final follow-up questionnaire was administered 3 months after the last training session.

### Measures

The questionnaires (shown in Additional file 2) were developed to measure mental health literacy, stigmatising attitudes, Mental Health First Aid behaviours, and the mental health and help-seeking status of adolescents. The questionnaires included items adapted from the Australian National Survey of Youth Mental Health Literacy and Stigma [23] and included a vignette depicting an adolescent (‘Jeanie’; see Table 2) experiencing social anxiety. All open-ended responses were coded by a member of the team who was blinded to measurement conditions, according to a structured protocol described elsewhere [24].

**Table 2:** Vignette used in student questionnaires

Jeanie is a 14 year old living at home with her parents. Jeanie started at your school last year and you are the only friend she has made so far. She seems very shy. When you ask her why she doesn’t make more of an effort, she says she would really like to make more friends but is scared that she’ll do or say something embarrassing when she’s around others. Although Jeanie’s schoolwork is OK she rarely says a word in class. She becomes incredibly nervous, trembles, blushes and seems like she might vomit if she has to answer a question or speak in front of the class. At her house you have seen that Jeanie is quite talkative with her family but becomes quiet if anyone she doesn’t know well comes over. She has stopped answering the phone and doesn’t come to parties anymore. Jeanie says she knows her fears are unreasonable but she can’t seem to control them and this really upsets her	

### Recognition of anxiety disorder

Problem recognition was assessed by asking students an open-ended question about what, if anything, was the matter with Jeanie. Responses were coded as correct if they mentioned social anxiety or social phobia, anxiety or an unspecified anxiety disorder. One researcher coded according to a validated coding frame [20] and blind to time-point. The labelling responses to the vignette have been previously validated against the diagnoses of mental health professionals [25], and have been found to predict both a preference for sources of help recommended by mental health professionals [26] and better-quality Mental Health First Aid responses [27].

### Quality of intended support

Students were provided with a list of 12 possible actions for responding to Jeanie and were asked to rate how likely they were to use these actions if Jeanie were a friend. Six of the possible actions were considered desirable as they were concordant with the tMHFA action plan of: Look for warning signs, Listen Up, Ask how they are, Help your friend connect with an adult, and Your friendship is important. Example items included: *Tell Jeanie I have noticed something seems wrong, and I want to make sure she is okay* and *Suggest Jeanie tell an adult (other than a health professional) about her problems (e.g. a parent or teacher).* The remaining six actions were discordant with the action plan and considered undesirable (e.g., *Ignore Jeanie because she is being attention*-*seeking* or *Let Jeanie know I won’t want to be friends with her any more if she’s like this all the time*). Students responded using a 5-point scale from ‘Definitely not’ to ‘Yes, definitely’. The undesirable responses were reverse scored. Two of the items were dropped because they lowered the reliability of the scale. The remaining 10 items were summed to give a total score ranging from 10 to 50, with higher scores indicating better quality of first aid intentions. Omega total was used to assess reliability [28], with values at pre-test of 0.74 (interval) and 0.83 (ordinal).

### Confidence in providing help

Confidence in helping Jeanie was rated in a 5-point scale from ‘Not at all confident’ to ‘Extremely confident’. In the control group of the previous randomised controlled trial evaluation of tMHFA 10–12, the test–retest reliability for this measure was *r *= 0.48 after 4 weeks. Previous evaluations of MHFA training in adults have shown this single item to reliably increase after provision of training [29].

### Number of adults thought to be helpful

Beliefs about help were assessed by asking students to rate a range of potential sources of help (close friend, counsellor, family member, general practitioner (GP) or family doctor, minister or priest, parent, psychologist, school counsellor/school well-being coordinator, teacher) as likely to be helpful, harmful or neither helpful or harmful, for Jeanie. These items were used to measure belief in accessing adult help, which is a key message of the training [17]. The students were given 1 point for each of the following rated as helpful: counsellor, GP, minister/priest, psychologist, school counsellor, teacher (scores range from 0 to 6).

### Appropriate help-seeking intentions

Students were asked to select which of 10 actions they would perform if they had a problem like Jeanie’s. Responses were scored by assigning 1 point for endorsing any of the following options, which were concordant with appropriate help-seeking as taught in the action plan: talk to a friend, talk to an adult, and talk to a health professional. Multiple responses were allowed and total scores ranged from 0 to 3.

### Stigmatising attitudes

Students were asked to respond to questions assessing personal stigma towards Jeanie, using a five-point Likert scale [30, 31]. Social distance was measured using five items adapted from a Social Distance Scale [30, 31]. The personal stigma and social distance items were used in combination to construct three stigma scales, which have previously been validated by exploratory structural equation modelling: weak-not-sick, dangerous/unpredictable and social distance [32]. There was also a single item (‘If I had a problem like Jeanie’s, I would not tell anyone’), which did not load on these factors and was scored separately. Omega total in the current sample at pre-test was 0.68 (interval) and 0.74 (ordinal) for weak-not-sick, 0.60 (interval) and 0.67 (ordinal) for dangerous/unpredictable, and 0.93 (interval) and 0.95 (ordinal) for social distance.

### Quality of support provided to a peer

Students’ experiences of providing support to a peer were assessed at baseline and at follow-up by asking if they had contact in the last 12 months (baseline) or in the last 3 months (follow-up) with anyone about their age who they thought might have a mental health problem or crisis, and whether they attempted to help them. If students responded ‘Yes’, they were asked whether they had given any of the 12 types of help listed in quality of intended support (see above), selecting all that applied. One point was given for each of six desirable first aid actions. Omega total at pre-test was 0.68 (interval) and 0.82 (ordinal).

### Quality of support received from a peer

Students were asked at baseline and at follow-up whether they had experienced a mental health problem or mental health crisis in the last 12 months (baseline) or three months (follow-up). If a student said ‘yes’ or ‘not sure’, they were asked further questions about whether they received any help, who provided it and what the person did to help, selecting from the same options as for help-seeking intentions above. If the support was provided by a peer, a count was made of the number of supportive items endorsed (out of 6). Omega total for this scale at pre-test was 0.58 (interval) and 0.72 (ordinal).

### Student mental health

Students’ mental health was assessed at baseline and follow-up using the K6. The K6 is a measure of psychological distress with possible scores ranging from 6 to 30, which has been validated against clinical diagnosis [33, 34]. Omega total (interval) for this scale at pre-test was 0.88.

### Participant satisfaction

In line with previous evaluations of MHFA training [19], course satisfaction was assessed immediately after the training using questions rated on 5-point Likert scales. These questions covered how new the information in the course was, how easy the information was to understand, how well the information was presented, how useful the information was and how useful the information would be in the future. Students also rated how much they liked the following parts of the program: PowerPoint presentation, student manual, videos and activities. Students were also asked to answer a series of open-ended questions about the strengths and weaknesses of the program, and how it could be improved. At follow-up, students were asked a series of questions about the manual and what they did with it. They were also asked whether they had talked about the program with anyone in their family.

Parents/guardians, student well-being staff and teachers were invited to complete a questionnaire three months after course completion, designed to qualitatively examine their perceptions of the course and students’ experience (see Additional file 2).

### Data analysis

#### Statistical analysis

The data were analysed with mixed models for continuous and binary outcome variables. This method is well suited to the data, as it takes into account its hierarchical structure, i.e. the correlation of measurement occasions within students and within schools. These maximum likelihood-based methods are able to produce unbiased estimates when a proportion of the participants withdraw before the completion of the study, based on the reasonable assumption that these data are missing at random. Models included a random effect for school clusters to adjust for the correlation of student responses within schools. Fixed effects were assessment point and age. Age was associated with missingness so was included as a fixed effect to help meet the missing at random assumption. School intraclass correlation coefficients (ICC) indicate the proportion of variability in the outcome attributable to school clusters.

Effect sizes (Cohen’s d) were calculated by dividing the difference between means by their pooled standard deviation. Analyses were performed in Stata 14 and Omegas were calculated using RStudio.

### Ethics, consent and permissions

Approval for the research was granted by the University of Melbourne Human Research Ethics Committee (ethics ID 1647390). Approval was also granted by the Victorian Department of Education and Early Childhood Development, and the Catholic Education Office Melbourne. For students at the Catholic school to be eligible to participate in the evaluation research, they required parental consent. All other students were eligible to participate unless their parents withdrew them from the study (opt-out consent). All students provided assent before completing surveys. Students with a known current mental health problem, previous experience of mental illness or suicide bereavement were encouraged to speak to their mental health professional, school counsellor or parents before deciding whether to participate.

## Results

### Participant characteristics and flow

There were 475 students with pre-test data who had a mean age of 13.86 years (SD = 0.45, range 12.07–15.15). The sample was 47.4% female, 51.6% male and 1.1% other, and 96.2% reported English as their first language.

**Fig. 2:**
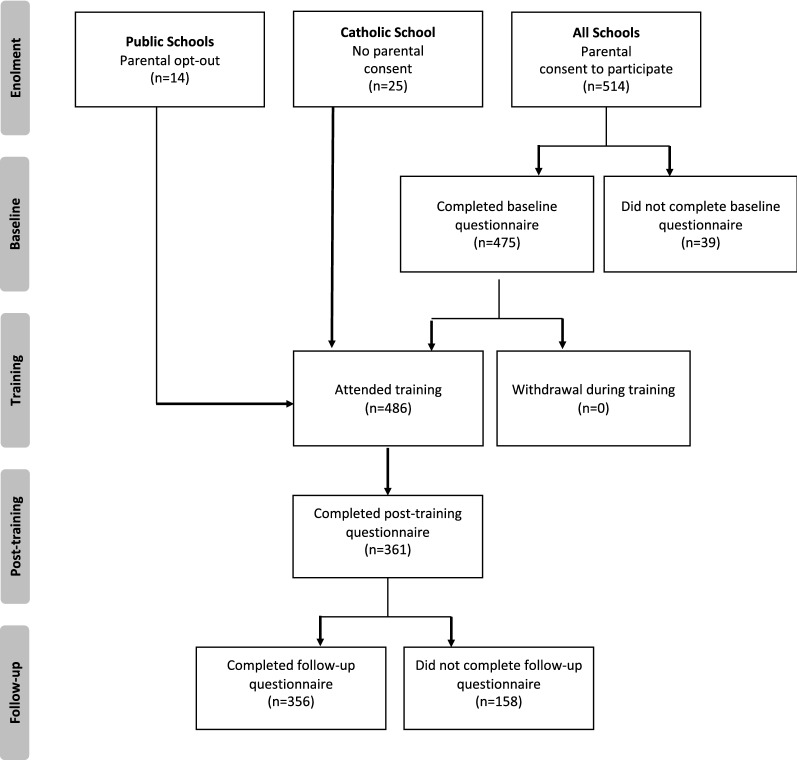
Participant flow diagram

Figure 2 shows the participant flow diagram. Of the students who had baseline data, 76.0% had post-test data, 75.0% had follow-up data and 69.1% had data at all three time points. A logistic regression analysis predicting attrition, adjusting for school clustering, found that two schools had higher attrition than the reference school (OR = 2.12, 95% CI 1.27–3.53, p = 0.004 and OR = 5.88, 95% CI 2.42–14.25, p < 0.001). There was also an effect of age (OR = 1.72, 95% CI 1.07–2.76, p = 0.026), but not of English as a first language, gender or K6 score.

### Changes over time in outcome measures

Table 3 shows the descriptive statistics for the continuous outcome measures and Table 4 shows the results of the associated mixed model analyses of change over time. Confidence in helping and quality of intended support improved from pre-test to post-test with small effect sizes, but these changes were not maintained at follow-up. ‘Stigma—weak not sick’ improved from pre-test to post-test and this was maintained at follow-up, although the effect size reduced from medium to small. Other aspects of stigma were less consistent. Social distance improved at post-test, with a small effect size, but this was not maintained at follow-up. On the other hand, ‘stigma—dangerous/unpredictable’ did not improve significantly at post-test, although there was a significant but very small improvement at follow-up. Quality of Mental Health First Aid provided to a peer improved from pre-test to follow-up with a small-to-medium effect size, but there was no significant change in quality of Mental Health First Aid received from peers.

**Table 3:** Descriptive statistics on the continuous outcome measures at each time point

	Observed means									Estimated marginal means					
	Pre			Post			3-month follow-up			Pre		Post		3-month follow-up	
	N	M	SD	N	M	SD	N	M	SD	M	SE	M	SE	M	SE
Confidence in helping Jeanie	472	3.75	0.97	354	3.91	0.94	353	3.81	0.94	3.74	0.04	3.90	0.05	3.79	0.05
Quality of intended support	470	40.77	5.09	351	42.20	5.80	352	40.95	5.85	40.68	0.36	41.97	0.38	40.70	0.38
Stigma—weak not sick	474	2.12	0.77	356	1.80	0.71	355	1.93	0.78	2.17	0.10	1.89	0.10	2.03	0.10
Stigma—dangerous/unpredictable	473	2.09	0.71	355	2.04	0.73	355	1.99	0.77	2.10	0.05	2.07	0.06	2.02	0.06
Social distance	475	1.80	0.67	360	1.66	0.65	356	1.73	0.68	1.80	0.04	1.69	0.04	1.75	0.04
Number of adults thought to be helpful for Jeanie	475	3.38	1.64	361	4.10	1.53	356	3.75	1.68	3.36	0.20	4.08	0.20	3.74	0.20
Quality of MHFA provided to peer	279	2.98	1.74				191	3.54	1.67	2.92	0.17			3.38	0.18
Quality of MHFA received from peer	85	2.98	1.55				63	3.29	1.73	2.96	0.18			3.26	0.21
K6	467	12.57	5.55				350	12.85	5.74	12.75	0.49			13.18	0.50

**Table 4:** Mixed model analyses of change over time for continuous outcome measures

	Main effect of time		Mean change over time contrasts									
			Pre to post					Pre to follow-up				
	p	School ICC	M	95% CI	p	d	95% CI	M	95% CI	p	d	95% CI
Confidence in helping Jeanie	0.003*	0.000	0.16	0.07 to 0.26	0.001*	0.17	0.03 to 0.31	0.05	− 0.04 to 0.15	0.267	0.07	− 0.07 to 0.21
Quality of intended support	< 0.001*	0.009	1.28	0.78 to 1.78	< 0.001*	0.26	0.12 to 0.40	0.02	− 0.49 to 0.52	0.952	0.03	− 0.11 to 0.17
Stigma—weak not sick	< 0.001*	0.076	− 0.28	− 0.34 to − 0.21	< 0.001*	0.43	0.29 to 0.56	− 0.14	− 0.20 to − 0.07	< 0.001*	0.24	0.10 to 0.38
Stigma—dangerous/unpredictable	0.093	0.013	− 0.02	− 0.09 to 0.05	0.511	0.07	− 0.07 to 0.20	− 0.08	− 0.15 to − 0.01	0.031*	0.13	− 0.00 to 0.27
Social distance	0.002	0.006	− 0.11	− 0.17 to − 0.05	< 0.001*	0.20	0.06 to 0.34	− 0.05	− 0.12 to 0.01	0.089	0.11	− 0.03 to 0.24
Number of adults helpful for Jeanie	< 0.001*	0.059	0.72	0.56 to 0.88	< 0.001*	0.45	0.31 to 0.59	0.38	0.22 to 0.54	< 0.001*	0.22	0.08 to 0.36
Quality of MHFA provided to peer	< 0.001*	0.027						0.47	0.23 to 0.70	< 0.001*	0.33	0.14 to 0.52
Quality of MHFA received from peer	0.239	0.001						0.30	− 0.20 to 0.80	0.239	0.19	− 0.14 to 0.52
K6	0.052^a^	0.024						0.43	− 0.00 to 0.87	0.052	− 0.05	− 0.19 to 0.09

*statistically significant

^a^This p-value was 0.194 after transforming the variable to adjust for positive skew associated with outliers

Table 5 shows the changes and mixed model results for the binary outcomes. The only significant change from pre-test to post-test was in whether the student would seek appropriate help, but this improvement was not maintained at follow-up. Correct recognition of the disorder in the vignette did not change from pre-test to post-test, but was significantly better at follow-up. Surprisingly, ‘stigma—would not tell anyone’ did not change from pre-test to post-test, but was significantly worse at follow-up.

**Table 5:** Descriptive statistics on the binary outcome measures at each time point and mixed model analyses of change over time

	Observed proportions						Estimated population-averaged proportions									
	Pre		Post		3-month follow-up		Pre	Post	3-month follow-up		Pre to post			Pre to follow-up		
	N	%	N	%	N	%	%	%	%	School ICC	OR	95% CI	p	OR	95% CI	p
Correct recognition of anxiety disorder	462	42.6	337	39.2	350	47.4	40.3	36.8	45.3	0.100	0.71	0.45–1.11	0.135	1.59	1.02–2.46	0.039*
Correct recognition of any mental health problem	462	49.8	337	54.3	350	52.6	47.4	51.2	50.2	0.065	1.34	0.89–2.03	0.163	1.25	0.83–1.87	0.284
Stigma—would not tell anyone (disagree/strongly disagree)	475	66.7	358	68.4	356	58.1	66.7	68.1	57.5	0.000	1.12	0.75–1.67	0.573	0.50	0.34–0.74	0.001*
Would seek appropriate help	475	82.5	361	88.1	356	84.6	82.0	87.6	83.5	0.036*	2.07	1.22–3.52	0.007*	1.19	0.72–1.96	0.493

*statistically significant

*ICC* intra-class correlation, *CI* confidence interval

### Participant satisfaction

Quantitative data on participant satisfaction is shown in Table 6. Students generally found the information in the course to be new, easy to understand, well presented and useful in the present and future. The videos were the most liked component of the course and the workbook the least liked.

**Table 6:** Participant satisfaction with the course

Question	Response option	Percentage (%)
How new was the information to you?	Not very new^a^	45.0
	Somewhat new	36.0
	Very new^a^	19.0
How hard was the information to understand?	Hard^a^	3.3
	Somewhat hard	15.6
	Easy^a^	81.0
How well was the course presented?	Not well^a^	9.8
	Somewhat well	29.4
	Well^a^	60.8
How useful was the information to you?	Not very useful^a^	28.8
	Somewhat useful	33.6
	Very useful^a^	37.5
How useful will the information from the course be in the future?	Not very useful^a^	16.5
	Somewhat useful	27.5
	Very useful^a^	56.0
Course components		
Powerpoint	Disliked	21
	Neutral	34
	Liked	45
Manual	Disliked	27
	Neutral	34
	Liked	39
Videos	Disliked^a^	11
	Neutral	20
	Liked^a^	70
Activities	Disliked^a^	22
	Neutral	25
	Liked^a^	53

^a^Collapsed from 2 points to 1

In response to open-ended questions, the students identified a number of strengths of the course, including that it gave them the practical skills to help their fellow students. In particular, the action plan was found to be a helpful tool. One student said that the course, “…made you feel that you could make a difference to someone’s life” and one well-being coordinator stated that staff had noticed students referring back to things they learned in the course. Other stated strengths were the films and the presentation skills of the Instructors. The main stated weakness of the course was that some students felt the course could have been more engaging by having additional activities and films. A few students also felt the course did not cater to students who started with a higher level of knowledge about mental health problems.

### Staff and parent satisfaction

Only 9 responses were received for the questionnaire to school staff (N = 5) and parents/guardians (N = 4). While the data are very limited, most of these adults thought that the students responded positively to the program and that it was useful for the students. There was variable opinion about how well the program was presented and perception of how much the students enjoyed it.

### Use of manual following course

Table 7 shows use of the manual following the course. Most students had read at least part of the manual and reported that it was neither easy nor difficult to understand. However, only minorities thought they would use it in the future, had kept it or had shown it to someone in their family.

**Table 7:** Participant use of the manual following the course

Question	Response option	Percentage (%)
How much of the manual did you read?	None of it	38.0
	Part of it	32.9
	Most of it	20.9
	All of it	8.0
How easy was it to understand?	Very difficult	1.2
	Difficult	1.7
	Neither easy not difficult	73.7
	Did not read	23.4
Do you think you will use the manual in the future?	Yes	11.4
	No	38.5
	Not sure	48.4
	I already have	1.7
What have you done with the manual?^a^	Kept it	29.5
	Lent it to someone	2.5
	Given it away	1.5
	Thrown it away	9.1
	Lost it	17.3
	Don’t know	23.4
Did you show the manual to anyone in your family?	Yes	13.8
	No	59.0
	Not sure/can’t remember	27.2

^a^Multiple responses could be selected

## Discussion

This evaluation found short-term improvements at the end of the training in confidence in providing help, quality of intended help, number of adults thought to be helpful, intentions to seek help for a mental health problem, social distance and some aspects of stigma. More importantly, there were sustained improvements over 3 months in number of adults thought to be helpful, some components of stigma, recognition of anxiety disorder and quality of support provided to a peer.

Comparing these findings to a previous evaluation of tMHFA 10–12 that had a similar uncontrolled design [19], the sustained effects for the tMHFA 7–9 program were not as strong as tMHFA 10–12 in the areas of confidence in providing help to others, social distance, willingness to tell others about a mental health problem and student mental health. The greatest difference between the outcomes of the current study and the evaluation of the tMHFA 10–12 program appeared on the measure of willingness to tell others, which showed a worsening at follow-up in the current study. By contrast, in the tMHFA 10–12 evaluation there were no changes in scores on this variable over time in response to the Jeanie vignette. In addition, in the 10–12 evaluation there was a significant improvement in scores on willingness to disclose in response to a vignette describing a young male with depression and suicidality (John).

There are a number of factors that may have contributed to fewer sustained changes with the tMHFA 7–9 course. The most obvious one is the age group of the students. It is possible that the topic is seen as less relevant by students in this age group, they are less developmentally equipped, or the pedagogical approach is less suited to younger age groups. However, student satisfaction ratings for tMHFA 7–9 were broadly similar to those of tMHFA 10–12, suggesting that students’ enjoyment of the course was not a major contributing factor to these findings. Another possible factor is that the tMHFA 10–12 evaluation used both a depression with suicidal thoughts vignette and a social anxiety vignette, whereas the current evaluation used only the social anxiety vignette. One reason for this was that pilot work showed that the younger students had more difficulty in completing the longer questionnaire with two vignettes, so one was removed to shorten it. Another was that the tMHFA 10–12 course included dealing with peers at risk of suicide, whereas this was not explicitly covered in the tMHFA 7–9 course. The suicide-related content and the use of the depression with suicidal thoughts vignette may have enabled greater changes, given the taboos around this topic.

However, these differences do not account for the finding that willingness to tell others about a mental health problem did not change from pre-test to post-test, but then worsened at follow-up. This pattern of change suggests that other factors may have been operating between the post-test and the follow-up questionnaires. One possibility that we are aware of is that the Netflix series *13 Reasons Why*, which deals with the events leading up to a young woman’s suicide, was released on 31 March 2017 to considerable publicity and controversy in Australia. The evaluation of the course ran from March to November 2017, so overlapped with the release of the series. Anecdotally, *13 Reasons Why* was widely viewed by adolescents in the region where tMHFA 7–9 training was carried out. This series negatively portrays the capacity of adults to intervene in a helpful way and research from other countries showed that the series can lead to worsening of mood and to suicide attempts [35, 36].

### Limitations

The main limitation of this study is a lack of a control group. This limitation was evident in our inability to control for external influences, such as the *13 Reasons Why* series. The uncontrolled design also meant that we could not control for any effects of repeated assessments. Nevertheless, the study provides the feasibility information necessary before embarking on a randomized controlled trial.

Another limitation was that we used only a social anxiety vignette, so that the effects of the training on students’ responses to other adolescent mental health problems are unknown.

## Conclusions

The study has shown that tMHFA 7–9 is acceptable to students and schools and that it has some positive effects that are maintained up to 3 months following training. It has also suggested some areas where the course needs to be further refined. Given that the risk of first onset of mental disorders is high throughout adolescence, training at one developmental point is unlikely to be sufficient. There may be merit in considering the two tMHFA courses as part of a package where the years 10–12 course boosts and extends the knowledge and skills of the years 7–9 course, and where parents and school staff are offered Youth MHFA training so that they learn to support any students who approach them about mental health problems.

## Supplementary information

**Additional file 1.** Sociodemographic characteristics of the schools.

**Additional file 2.** Surveys: baseline (T1), post-training (T2), follow-up (T3) and parent/guardian and teacher feedback survey.

## Data Availability

The datasets used and/or analysed during the current study are available from the corresponding author on reasonable request. All surveys used are also available upon request to the authors.

## Abbreviations

MHFA: Mental Health First Aid
tMHFA 10-12: Teen MHFA course for students in years 10 to 12 of secondary school
tMHFA 7-9: Teen MHFA course for students in years 7 to 9 of secondary school
OR: odds ratio
