# Supplementary material for: Teen Mental Health First Aid for years 7–9: a description of the program and an initial evaluation
Source: Int J Ment Health Syst. 2019 Nov 16;13:71. doi: 10.1186/s13033-019-0325-4 (PMC6858708; doi:10.1186/s13033-019-0325-4)
Supplement: Supplementary file 1 — Additional file 1. Sociodemographic characteristics of the schools. [file 13033_2019_325_MOESM1_ESM.pdf]

Additional file 1: Sociodemographic characteristics of the schools

Characteristic	School 1	School 2	School 3	School 4	School 5
**Location**	Regional	Metropolitan	Metropolitan	Metropolitan	Metropolitan
**School sector**	Non-government	Government	Government	Government	Government
**Year range**	7-12	7-12	7-12	7-12	7-12
**ICSEA***	1062	1141	940	986	965
**Total enrolments**	805	453	551	887	281
**Percent girls**	52	49	47	47	48
**Percent language other than English**	5	30	5	7	2
**Percent Indigenous**	1	1	5	1	2

* ICSEA = Index of Socio-Educational Advantage, with M=1000 and SD=100
