# Supplementary material for: Teen Mental Health First Aid for years 7–9: a description of the program and an initial evaluation
Source: Int J Ment Health Syst. 2019 Nov 16;13:71. doi: 10.1186/s13033-019-0325-4 (PMC6858708; doi:10.1186/s13033-019-0325-4)
Supplement: Supplementary file 2 — Additional file 2. Surveys: baseline (T1), post-training (T2), follow-up (T3) and parent/guardian and teacher feedback survey. [file 13033_2019_325_MOESM2_ESM.pdf]

Evaluating the teen Mental Health First Aid course for students in years 7-9
(Survey T1)

Information about you

1. Please enter your student identification number (e.g. AND0001).

[ ]

2. Would you like to do the survey?

- ○ Yes, I have read and understood this information and am willing to proceed.
- ○ No, I do not want to do this survey. (If you tick 'No', please do not complete any more of the survey.)

3. What is your age?

- ○ 12
- ○ 13
- ○ 14
- ○ 15

4. How many months since your last birthday?

- ○ 0
- ○ 1
- ○ 2
- ○ 3
- ○ 4
- ○ 5
- ○ 6
- ○ 7
- ○ 8
- ○ 9
- ○ 10
- ○ 11

5. What is your gender?

- ○ Male
- ○ Female
- ○ I identify with another term

6. Is English your first language? (By this we mean is English the language you speak at home with your family?)

- ○ Yes
- ○ No

7. Will you be 14 or older in 2017?

- ○ Yes
- ○ No

Jeanie's story

Please read Jeanie's story carefully, as the following questions relate to it.

Jeanie is a 14 year old living at home with her parents. Jeanie started at your school last year and you are the only friend she has made so far. She seems very shy. When you ask her why she doesn't make more of an effort, she says she would really like to make more friends but is scared that she'll do or say something embarrassing when she's around others. Although Jeanie's schoolwork is OK she rarely says a word in class. She becomes incredibly nervous, trembles, blushes and seems like she might vomit if she has to answer a question or speak in front of the class. At her house you have seen that Jeanie is quite talkative with her family, but becomes quiet if anyone she doesn't know well comes over. She has stopped answering the phone and doesn't come to parties anymore. Jeanie says she knows her fears are unreasonable but she can't seem to control them and this really upsets her.

8. What, if anything, do you think is the matter with Jeanie?

9. If Jeanie was a friend, I would... (Tick your answer.)

	**Definitely not**	**Probably not**	**Not sure**	**Yes, probably**	**Yes, definitely**
Tell Jeanie what she needs to do to fix her problems.					
Invite Jeanie to hang out and do something fun with me.					
Ignore Jeanie because she is being attention-seeking.					
Let Jeanie know I won't want to be friends with her any more if she's like this all the time.					
Avoid talking about suicide, because it might put the idea in Jeanie's head.					
Tell Jeanie I have noticed something seems wrong, and I want to make sure she is okay.					
Suggest Jeanie tell a health professional about her problems (e.g. a counsellor, GP or psychologist).					
Suggest Jeanie tell an adult (other than a health professional) about her problems (e.g. a parent or teacher).					
Ask Jeanie if she is thinking of suicide.					
Encourage Jeanie to take responsibility and deal with her problems on her own.					
Listen to Jeanie talk about her problems.					
Not do anything.					
Other, please specify:					

10. If Jeanie was a friend, how confident would you feel in helping her? (Circle your answer.)

Not at all confident	A little bit confident	Moderately confident	Quite a bit confident	Extremely confident

11. Which of the following do you think would be helpful, harmful, or neither helpful or harmful for Jeanie 's problem? (Tick your answer.)

	Helpful	Neither	Harmful
Close friend			
Counsellor			
Family member			
General practitioner (GP) or family doctor			
Minister or priest			
Parent			
Psychologist			
School counsellor/School wellbeing coordinator			
Teacher			

12. If I had a problem right now like Jeanie's, I would... (Please note you can select **more than one** option.)

- ○ Try to deal with it on my own.
- ○ Talk to a friend about it.
- ○ Talk to a health professional about it (e.g. a counsellor, GP or psychologist).
- ○ Talk to an adult (other than a health professional) about it (e.g. a parent or teacher).
- ○ Improve my diet.
- ○ Try to get more sleep.
- ○ Spend more time with friends.
- ○ Try to do more exercise.
- ○ Look for information about my problem on the internet.
- ○ Do nothing.
- ○ Other, please specify:

13. Please indicate how strongly you agree or disagree with each statement. (Tick your answer.)

	**Strongly disagree**	**Disagree**	**Neither agree nor disagree**	**Agree**	**Strongly agree**
A problem like Jeanie's is a sign of personal weakness.					
If I had a problem like Jeanie's, I would not tell anyone.					
It is best to avoid people with a problem like Jeanie's so that you don't develop this problem.					
Jeanie's problem is not a real medical illness.					
People with a problem like Jeanie's could snap out of it if they wanted.					
People with a problem like Jeanie's are unpredictable.					
People with a problem like Jeanie's are dangerous.					

14. The following questions ask how you would feel about spending time with Jeanie. Would you be happy to... (Tick your answer.)

	**Yes, definitely**	**Yes, probably**	**Probably not**	**Definitely not**
Develop a close friendship with Jeanie?				
Go out with Jeanie on the weekend?				
Go to Jeanie's house?				
Invite Jeanie around to your house?				
Work on a project with Jeanie?				

<u>Experience of mental health problems</u>

This section asks some questions about your experiences of mental health problems. Please read each question carefully and answer any questions when they apply.

Definitions
A <u>mental health problem</u> is when there is a major change in a person's normal way of thinking, feeling or behaving. It affects the person's ability to get on with life. It does not go away quickly or lasts longer than normal emotions or reactions would be expected to. It might involve a diagnosed mental illness, a worsening of mental health or an undiagnosed problem, or a drug or alcohol problem.

A <u>mental health crisis</u> is when a person is at increased risk of harm to themselves or to others. Crisis situations include having thoughts of suicide, engaging in self-injury, being very intoxicated with alcohol or other drugs, or experiencing bullying or abuse.

15. In the last 12 months, have you had contact with anyone about your age (between 12 and 18 years old) who you thought might have a mental health problem or has experienced a mental health crisis?
Please do not report contact with adults. For this question, we are just interested in people around your age who you may have helped.

- ○ Yes (Please go to question 16 below)
- ○ No (Please go to question 20 on page 13)
- ○ Not sure (Please go to question 16 below)
- ○ I don't want to answer the question (Please go to question 20 on page 13)

<u>Help you have given</u>

16. Please tell us how many people about your age you had contact with who you thought might have a mental health problem or experienced a mental health crisis:

- ○ 1
- ○ 2
- ○ 3 or more

If you have had contact with more than one person, please answer the following questions about <u>the person who you know best.</u> If you only had contact with one person, please answer the following questions about that person.

17. Did you offer the person any help?

- ○ Yes (Please go to question 18 below)
- ○ No (Please go to question 19 on page 13)
- ○ Not sure (Please go to question 18 below)

18. What did you do to help the person? (**Choose all that apply**.) *AFTER ANSWERING THIS QUESTION SKIP TO QUESTION 20.*

- ○ Told them what they needed to do to fix their problems.
- ○ Invited them to hang out and do something fun with me.
- ○ Ignored them because they were being attention-seeking.
- ○ Let them know I didn't want to be friends with them any more if they would be like this all the time.
- ○ Avoided talking about suicide, because it might put the idea in their head.
- ○ Told them I have noticed something seems wrong, and I wanted to make sure they were okay.
- ○ Suggested they tell a health professional about their problems (e.g. a counsellor, GP or psychologist).
- ○ Suggested they tell an adult (other than a health professional) about their problems (e.g. a parent or teacher).
- ○ Asked them if they were thinking of suicide.
- ○ Encouraged them to take responsibility and deal with their problems on their own.
- ○ Listened to them talk about their problems.
- ○ I didn't do anything.
- ○ Other, please specify:

Unable to help

19. Why were you unable to help the person?

Your own mental health

20. In the last 12 months, have you had a mental health problem or experienced a mental health crisis? Please report any mental health problem you think you might have had, whether or not it has been diagnosed by a doctor or mental health professional.

- ○ Yes (Please go to question 21 below)
- ○ No (Please go to question 24 on page 15)
- ○ Not sure (Please go to question 21 below)
- ○ I don't want to answer this question (Please go to question 24 on page 15)

21. In the last 12 months, has anyone tried to support or help you with this mental health problem or mental health crisis?

- ○ Yes (Please go to question 22 on page 14)
- ○ No (Please go to question 24 on page 15)
- ○ Not sure (Please go to question 22 on page 14)

Help you have received

22. Who provided support or help for the problem? (Please note you can select **more than one option**.)

- ○ Friend (Please go to question 23 below)
- ○ Parent (Please go to question 24 on page 15)
- ○ Other family member (Please go to question 24 on page 15)
- ○ Health professional, e.g. a counsellor, GP or psychologist (Please go to question 24 on page 15)
- ○ Teacher (Please go to question 24 on page 15)
- ○ Other, please specify: (write in your answer then go to question 24 on page 15)

Help you have received from your friend

ONLY ANSWER THIS QUESTION IF YOU TICKED 'FRIEND' IN QUESTION 22.

23. What did your friend do to help you? Please note you can select more than one option.

- ○ Told me what I needed to do to fix my problems.
- ○ Invited me to hang out and do something fun with them.
- ○ Ignored me because they thought I was being attention-seeking.
- ○ Let me know they didn't want to be friends with me any more if I would be like this all the time.
- ○ Told me they had noticed something seems wrong, and they wanted to make sure I was okay.
- ○ Suggested I tell a health professional about my problems (e.g. a counsellor, GP or psychologist).
- ○ Suggested I tell an adult (other than a health professional) about my problems (e.g. a parent or teacher).
- ○ Asked me if I was thinking of suicide.
- ○ Encouraged me to take responsibility and deal with my problems on my own.
- ○ Listened to me talk about my problems.
- ○ Other, please specify:

How you are feeling

24. In the past 30 days, about how often did you feel…

	None of the time	A little of the time	Some of the time	Most of the time	All of the time
Nervous?					
Hopeless?					
Restless or fidgety?					
So depressed that nothing could cheer you up?					
That everything was an effort?					
Worthless?					

<u>This is the end of the survey</u>

You can tear this page off and take it with you.

If you are feeling distressed at any stage and would like some support, please try talking to your school wellbeing coordinator. You could also try talking to your homeroom teacher or parents.

You could also try one of the following free services:

Kids Helpline: Phone 1800 55 1800 or visit kidshelp.com.au

Lifeline: Phone 13 11 14 or visit lifeline.org.au

ReachOut: Visit au.reachout.com

Evaluating the teen Mental Health First Aid course for students in years 7-9
(Survey T2)

Information about you

1. Please enter your student identification number (e.g. AND0001).

2. Would you like to do the survey?

- ○ Yes, I have read and understood this information and would like to proceed.
- ○ No, I do not want to do this survey. (If you tick 'No', please do not complete any more of the survey.)

Jeanie's story

Please read Jeanie's story carefully, as the following questions relate to it.

Jeanie is a 14 year old living at home with her parents. Jeanie started at your school last year and you are the only friend she has made so far. She seems very shy. When you ask her why she doesn't make more of an effort, she says she would really like to make more friends but is scared that she'll do or say something embarrassing when she's around others. Although Jeanie's schoolwork is OK she rarely says a word in class. She becomes incredibly nervous, trembles, blushes and seems like she might vomit if she has to answer a question or speak in front of the class. At her house you have seen that Jeanie is quite talkative with her family, but becomes quiet if anyone she doesn't know well comes over. She has stopped answering the phone and doesn't come to parties anymore. Jeanie says she knows her fears are unreasonable but she can't seem to control them and this really upsets her.

3. What, if anything, do you think is the matter with Jeanie?

4. If Jeanie was a friend, I would... (Tick your answer.)

	**Definitely not**	**Probably not**	**Not sure**	**Yes, probably**	**Yes, definitely**
Tell Jeanie what she needs to do to fix her problems.					
Invite Jeanie to hang out and do something fun with me.					
Ignore Jeanie because she is being attention-seeking.					
Let Jeanie know I won't want to be friends with her any more if she's like this all the time.					
Avoid talking about suicide, because it might put the idea in Jeanie's head.					
Tell Jeanie I have noticed something seems wrong, and I want to make sure she is okay.					
Suggest Jeanie tell a health professional about her problems (e.g. a counsellor, GP or psychologist).					
Suggest Jeanie tell an adult (other than a health professional) about her problems (e.g. a parent or teacher).					
Ask Jeanie if she is thinking of suicide.					
Encourage Jeanie to take responsibility and deal with her problems on her own.					
Listen to Jeanie talk about her problems.					
Not do anything.					
Other, please specify:					

5. If Jeanie was a friend, how confident would you feel in helping her? (Circle your answer.)

**Not at all confident**	**A little bit confident**	**Moderately confident**	**Quite a bit confident**	**Extremely confident**

6. Which of the following do you think would be helpful, neither helpful or harmful or harmful, for Jeanie 's problem? (Tick your answer.)

	**Helpful**	**Neither**	**Harmful**
Close friend			
Counsellor			
Family member			
General practitioner (GP) or family doctor			
Minister or priest			
Parent			
Psychologist			
School counsellor/School wellbeing coordinator			
Teacher			

7. If I had a problem right now like Jeanie's, I would... (Please note you can select **more than one** option.)

- ○ Try to deal with it on my own.
- ○ Talk to a friend about it.
- ○ Talk to a health professional about it (e.g. a counsellor, GP or psychologist).
- ○ Talk to an adult (other than a health professional) about it (e.g. a parent or teacher).
- ○ Improve my diet.
- ○ Try to get more sleep.
- ○ Spend more time with friends.
- ○ Try to do more exercise.
- ○ Look for information about my problem on the internet.
- ○ Do nothing.
- ○ Other, please specify:

8. Please indicate how strongly you agree or disagree with each statement. (Tick your answer.)

	**Strongly disagree**	**Disagree**	**Neither agree nor disagree**	**Agree**	**Strongly agree**
A problem like Jeanie's is a sign of personal weakness.					
If I had a problem like Jeanie's, I would not tell anyone.					
It is best to avoid people with a problem like Jeanie's so that you don't develop this problem.					
Jeanie's problem is not a real medical illness.					
People with a problem like Jeanie's could snap out of it if they wanted.					
People with a problem like Jeanie's are unpredictable.					
People with a problem like Jeanie's are dangerous.					

9. The following questions ask how you would feel about spending time with Jeanie. Would you be happy to... (Tick your answer.)

	**Yes, definitely**	**Yes, probably**	**Probably not**	**Definitely not**
Develop a close friendship with Jeanie?				
Go out with Jeanie on the weekend?				
Go to Jeanie's house?				
Invite Jeanie around to your house?				
Work on a project with Jeanie?				

<u>Your thoughts about tMHFA program</u>

10. How many tMHFA classroom sessions did you attend? Please do not include survey sessions in this answer. We are only interested in the number of sessions you attended which had an instructor present.

- ○ 0
- ○ 1
- ○ 2
- ○ 3 (all sessions)
- ○ I don't know/I can't remember

11. How new was this information in the program to you? (Please circle your answer.)

**Not new at all** (1)	2	3	4	**Very new** (5)

12. How easy was the information in the program to understand? (Please circle your answer.)

**Very hard** (1)	2	3	4	**Very easy** (5)

13. How well was the program presented? (Please circle your answer.)

**Very badly** (1)	2	3	4	**Very well** (5)

14. How useful was the program to you? (Please circle your answer.)

**Not very useful** (1)	2	3	4	**Very useful** (5)

15. How useful do you think the program's information will be for you **in the future**? (Please circle your answer.)

**Not very useful** (1)	2	3	4	**Very useful** (5)

16. Please rate how much you liked the following parts of the program:

	**Disliked** (1)	2	3	4	**Liked** (5)
PowerPoint presentation					
Student manual					
Videos					
Activities					

17. What do you think where the strengths of the program?

18. What do you think were the weaknesses of the program?

19. What would you do to make the program better?

20. Is there anything else you would like to say about the program?

21. Did any of the information provided in the program make you feel distressed (e.g. sad, stressed, worried, overwhelmed or nervous?)

- ○ No (You do not need to answer the remaining questions)
- ○ Not sure
- ○ Yes

<u>If you felt distressed</u>

22. Please describe what information in particular made you feel distressed?

23. If you felt distressed, would say that it was still worthwhile attending the tMHFA program? (Please circle your answer.)

Definitely not	Probably not	Not sure	Yes, probably	Yes, definitely

24. How long did the distressed feelings last for?

- ○ A few moments
- ○ A few hours
- ○ A few days
- ○ A week or more

25. Do you want someone to contact you to talk to you about how you are feeling, or about the tMHFA program?

- ○ Yes
- ○ No

26. Who would you like to contact you?

- ○ I do not want to be contacted
- ○ My regular homeroom teacher
- ○ My school counsellor/school wellbeing coordinator
- ○ My tHMFA program instructor or a member of the research team.

<u>This is the end of the survey</u>

You can tear this page off and take it with you.

If you are feeling distressed at any stage and would like some support, please try talking to your school wellbeing coordinator. You could also try talking to your homeroom teacher or parents.

You could also try one of the following free services:

Kids Helpline: Phone 1800 55 1800 or visit kidshelp.com.au

Lifeline: Phone 13 11 14 or visit lifeline.org.au

ReachOut: Visit au.reachout.com

Evaluating the teen Mental Health First Aid course for students in years 7-9

(Survey T3)

Information about you

1. Please enter your student identification number (e.g. AND0001).

2. Would you like to do the survey?

- ○ Yes, I have read and understood this information and would like to proceed.
- ○ No, I do not want to do this survey. (If you tick 'No', please do not complete any more of the survey.

Jeanie's story

Please read Jeanie's story carefully, as the following questions relate to it.

Jeanie is a 14 year old living at home with her parents. Jeanie started at your school last year and you are the only friend she has made so far. She seems very shy. When you ask her why she doesn't make more of an effort, she says she would really like to make more friends but is scared that she'll do or say something embarrassing when she's around others. Although Jeanie's schoolwork is OK she rarely says a word in class. She becomes incredibly nervous, trembles, blushes and seems like she might vomit if she has to answer a question or speak in front of the class. At her house you have seen that Jeanie is quite talkative with her family, but becomes quiet if anyone she doesn't know well comes over. She has stopped answering the phone and doesn't come to parties anymore. Jeanie says she knows her fears are unreasonable but she can't seem to control them and this really upsets her.

3. What, if anything, do you think is the matter with Jeanie?

4. If Jeanie was a friend, I would... (Tick your answer.)

	**Definitely not**	**Probably not**	**Not sure**	**Yes, probably**	**Yes, definitely**
Tell Jeanie what she needs to do to fix her problems.					
Invite Jeanie to hang out and do something fun with me.					
Ignore Jeanie because she is being attention-seeking.					
Let Jeanie know I won't want to be friends with her any more if she's like this all the time.					
Avoid talking about suicide, because it might put the idea in Jeanie's head.					
Tell Jeanie I have noticed something seems wrong, and I want to make sure she is okay.					
Suggest Jeanie tell a health professional about her problems (e.g. a counsellor, GP or psychologist).					
Suggest Jeanie tell an adult (other than a health professional) about her problems (e.g. a parent or teacher).					
Ask Jeanie if she is thinking of suicide.					
Encourage Jeanie to take responsibility and deal with her problems on her own.					
Listen to Jeanie talk about her problems.					
Not do anything.					
Other, please specify:					

5. If Jeanie was a friend, how confident would you feel in helping her? (Circle your answer.)

**Not at all confident**	**A little bit confident**	**Moderately confident**	**Quite a bit confident**	**Extremely confident**

6. Which of the following do you think would be helpful, harmful, or neither helpful or harmful for Jeanie 's problem?

	**Helpful**	**Neither**	**Harmful**
Close friend			
Counsellor			
Family member			
General practitioner (GP) or family doctor			
Minister or priest			
Parent			
Psychologist			
School counsellor/School wellbeing coordinator			
Teacher			

7. If I had a problem right now like Jeanie's, I would... (Please note you can select **more than one** option.)

- ○ Try to deal with it on my own.
- ○ Talk to a friend about it.
- ○ Talk to a health professional about it (e.g. a counsellor, GP or psychologist).
- ○ Talk to an adult (other than a health professional) about it (e.g. a parent or teacher).
- ○ Improve my diet.
- ○ Try to get more sleep.
- ○ Spend more time with friends.
- ○ Try to do more exercise.
- ○ Look for information about my problem on the internet.
- ○ Do nothing.
- ○ Other, please specify:

8. Please indicate how strongly you agree or disagree with each statement.

	**Strongly disagree**	**Disagree**	**Neither agree nor disagree**	**Agree**	**Strongly agree**
A problem like Jeanie's is a sign of personal weakness.					
If I had a problem like Jeanie's, I would not tell anyone.					
It is best to avoid people with a problem like Jeanie's so that you don't develop this problem.					
Jeanie's problem is not a real medical illness.					
People with a problem like Jeanie's could snap out of it if they wanted.					
People with a problem like Jeanie's are unpredictable.					
People with a problem like Jeanie's are dangerous.					

9. The following questions ask how you would feel about spending time with Jeanie. Would you be happy to... (Tick your answer.)

	**Yes, definitely**	**Yes, probably**	**Probably not**	**Definitely not**
Develop a close friendship with Jeanie?				
Go out with Jeanie on the weekend?				
Go to Jeanie's house?				
Invite Jeanie around to your house?				
Work on a project with Jeanie?				

Experience of mental health problems

This section asks some questions about your experiences of mental health problems. Please read each question carefully and answer any questions when they apply.

Definitions
A mental health problem is when there is a major change in a person's normal way of thinking, feeling or behaving. It affects the person's ability to get on with life. It does not go away quickly or lasts longer than normal emotions or reactions would be expected to. It might involve a diagnosed mental illness, a worsening of mental health or an undiagnosed problem, or a drug or alcohol problem.

A mental health crisis is when a person is at increased risk of harm to themselves or to others. Crisis situations include having thoughts of suicide, engaging in self-injury, being very intoxicated with alcohol or other drugs, or experiencing bullying or abuse.

10. In the last 3 months, have you had contact with anyone about your age (between 12 and 18 years old) who you thought might have a mental health problem or has experienced a mental health crisis?
Please do not report contact with adults. For this question, we are just interested in people around your age who you may have helped.

- ○ Yes (Please go to question 11 below)
- ○ No (Please go to question 21 on page 13)
- ○ Not sure (Please go to question 11 below)
- ○ I don't want to answer the question (Please go to question 21 on page 13)

Help you have given

11. Please tell us how many people about your age you had contact with who you thought might have a mental health problem or experienced a mental health crisis:

- ○ 1
- ○ 2
- ○ 3 or more

If you have had contact with more than one person, please answer the following questions about the person who you know best. If you only had contact with one person, please answer the following questions about that person.

12. Did you offer the person any help?

- ○ Yes (Please go to question 13 below)
- ○ No (Please go to question 20 on page 13)
- ○ Not sure (Please go to question 13 below)

13. What did you do to help the person? (Choose all that apply.)

- ○ Told them what they needed to do to fix their problems.
- ○ Invited them to hang out and do something fun with me.
- ○ Ignored them because they were being attention-seeking.
- ○ Let them know I didn't want to be friends with them any more if they would be like this all the time.
- ○ Avoided talking about suicide, because it might put the idea in their head.
- ○ Told them I have noticed something seems wrong, and I wanted to make sure they were okay.
- ○ Suggested they tell a health professional about their problems (e.g. a counsellor, GP or psychologist).
- ○ Suggested they tell an adult (other than a health professional) about their problems (e.g. a parent or teacher).
- ○ Asked them if they were thinking of suicide.
- ○ Encouraged them to take responsibility and deal with their problems on their own.
- ○ Listened to them talk about their problems.
- ○ I didn't do anything.
- ○ Other, please specify:

14. If you suggested that the person you were helping talk to an adult, did they talk to an adult?

- ○ Yes
- ○ No
- ○ Not sure
- ○ They were already being helped by an adult
- ○ I did not suggest they talk to an adult

15. When helping the person, did you use any information from the tMHFA program?

- ○ Yes
- ○ No
- ○ Not sure

16. When helping the person, did you do anything differently from what you would have done before attending the tMHFA program?

- ○ No
- ○ Not sure
- ○ Yes- Please briefly explain what you did differently:

17. Do you think what you did helped the person? (Please circle your answer.)

No, it was very unhelpful	No, it was unhelpful	I'm not sure	Yes, it was helpful	Yes, it was very helpful

18. Would you like to comment on what happened? (You do not have to answer this question.)

19. Do you think the information in the tMHFA program contributed to how helpful your assistance was? (Please circle your answer.)

Definitely not	Probably not	Not sure	Yes, probably	Yes, definitely

Unable to help

Only answer this question if you answered 'No' to question 12.

20. Why were you unable to help the person?

Your own mental health

21. In the last 3 months, have you had a mental health problem or experienced a mental health crisis? Please report any mental health problem you think you might have had, whether or not it has been diagnosed by a doctor or mental health professional.

- ○ Yes (Please go to question 22 below)
- ○ No (Please go to question 25 on page 14)
- ○ Not sure (Please go to question 22 below)
- ○ I don't want to answer this question (Please go to question 25 on page 14)

22. In the last 3 months, has anyone tried to support or help you with this mental health problem or mental health crisis?

- ○ Yes (Please go to question 23 below)
- ○ No (Please go to question 25 on page 14)
- ○ Not sure (Please go to question 23 below)

Help you have received

23. Who provided support or help for the problem? (Please note you can select **more than one option**.)

- ○ Friend
- ○ Parent
- ○ Other family member
- ○ Health professional (e.g. a counsellor, GP or psychologist)
- ○ Teacher
- ○ Other, please specify:

<u>Help you have received from your friend</u>

(Only answer this if ticked 'friend' in question 23)

24. What did your friend do to help you? Please note you can select more than one option.

- ○ Told me what I needed to do to fix my problems.
- ○ Invited me to hang out and do something fun with them.
- ○ Ignored me because they thought I was being attention-seeking.
- ○ Let me know they didn't want to be friends with me any more if I would be like this all the time.
- ○ Told me they had noticed something seems wrong, and they wanted to make sure I was okay.
- ○ Suggested I tell a health professional about my problems (e.g. a counsellor, GP or psychologist).
- ○ Suggested I tell an adult (other than a health professional) about my problems (e.g. a parent or teacher).
- ○ Asked me if I was thinking of suicide.
- ○ Encouraged me to take responsibility and deal with my problems on my own.
- ○ Listened to me talk about my problems.
- ○ I didn't do anything.
- ○ Other, please specify:

<u>Actions following the program</u>

This section contains questions about the tMHFA manual and about actions taken after finishing the tMHFA program.

25. How much of the tMHFA manual did you read after completing the tMHFA program? (Please circle your answer.)

**None of it**	**Part of it**	**Most of it**	**All of it**

26. If you read the manual, how easy was it to understand? (Please circle your answer.)

**Very difficult**	**Difficult**	**Neither easy nor difficult**	**Easy**	**Very easy**	**I did not read the manual**

27. Do you think you will use the manual in the future?

- ○ Yes
- ○ No
- ○ Not sure
- ○ I already have

28. What have you done with the manual? (Please note you can select **more than one option**.)

- ○ Kept it
- ○ Lent it to someone
- ○ Given it away
- ○ Thrown it away
- ○ Lost it
- ○ Don't know

29. Did you show the manual to anyone in your family?

- ○ Yes
- ○ No
- ○ Not sure/I can't remember

30. Did you talk about the tMHFA program with anyone in your family?

- ○ No
- ○ Yes- Please briefly tell us what you spoke about:

31. Did anyone in your family complete the Youth Mental Health First Aid course offered to parents/guardians?

- ○ Yes
- ○ No
- ○ Not sure

32. Is there anything else you would like to say about the tMHFA program, e.g. its strengths and weaknesses, how you would make it better?

How you are feeling

33. In the past 30 days, about how often did you feel…

	**None of the time**	**A little of the time**	**Some of the time**	**Most of the time**	**All of the time**
Nervous?					
Hopeless?					
Restless or fidgety?					
So depressed that nothing could cheer you up?					
That everything was an effort?					
Worthless?					

<u>This is the end of the survey</u>

You can tear this page off and take it with you.

If you are feeling distressed at any stage and would like some support, please try talking to your school wellbeing coordinator. You could also try talking to your homeroom teacher or parents.

You could also try one of the following free services:

Kids Helpline: Phone 1800 55 1800 or visit kidshelp.com.au

Lifeline: Phone 13 11 14 or visit lifeline.org.au

ReachOut: Visit au.reachout.com

tMHFA 7-9 - Parent/Guardian and Teacher Feedback Survey

Consent

This is about whether you agree to participate in the survey.

* 1. Would you like to proceed with the survey?

- ◯ Yes, I have read and understood the information provided to me and would like to proceed.
- ◯ No, I do not want to complete this survey.

tMHFA 7-9 - Parent/Guardian and Teacher Feedback Survey

Demographic information

This page contains some questions about you.

* 2. Which of the following describes your involvement with the tMHFA evaluation?

- ◯ I am a parent/guardian of a student who received the tMHFA program
- ◯ I am a teacher of students who received the tMHFA program
- ◯ I am a member of the student wellbeing staff/I am a school counsellor
- ◯ Other (please specify)

* 3. Have you ever attended a Mental Health First Aid course?
Examples include the Standard, Youth and Aboriginal and Torres Strait Islander Mental Health First Aid courses.

- ◯ Yes
- ◯ No
- ◯ Not sure

* 4. What is your age in years?

* 5. What is your gender?

- ◯ Male
- ◯ Female
- ◯ I identify with another term

* 6. Is English your first language? (By this we mean is English the language you speak at home with your family?)

- ◯ Yes
- ◯ No

tMHFA 7-9 - Parent/Guardian and Teacher Feedback Survey

Responses to the tMHFA program

This page contains questions about the tMHFA program and how you think we can improve it.

* 7. Have you spoken to your child/the students about the tMHFA program and its contents?

- Yes
- No
- Not sure

* 8. Do you feel like you have a good understanding of what was presented and discussed in the tMHFA program?

- Yes
- No
- Not sure

* 9. How do you think your child/the students responded to the program?

Very negatively (1)	2	3	4	Very positively (5)	Not sure
◯	◯	◯	◯	◯	◯

* 10. How useful do you think the information in the program was for your child/the students?

Not at all useful (1)	2	3	4	Very useful (5)	Not sure
◯	◯	◯	◯	◯	◯

* 11. How well do you think the program was presented to your child/the students?

Very badly (1)	2	3	4	Very well (5)	Not sure
◯	◯	◯	◯	◯	◯

* 12. How much do you think your child/the students enjoyed the program?

Not very much (1)	2	3	4	Very much (5)	Not sure
◯	◯	◯	◯	◯	◯

tMHFA 7-9 - Parent/Guardian and Teacher Feedback Survey

Responses to the tMHFA program

This page contains some questions about your thoughts on the program.

* 13. From <u>your</u> perspective, what do you think were the strengths of the program?

* 14. From <u>your child's/the students'</u> perspective, what do you think were the strengths of the program?

* 15. From <u>your</u> perspective, what do you think were the weaknesses of the program?

* 16. From <u>your child's/the students'</u> perspective, what do you think were the weaknesses of the program?

* 17. Do you think your child/the students will use the information presented to them in the program?

- ○ Yes
- ○ No
- ○ Not sure
- ○ I know they have already used the information.

If they have already used the information, please briefly describe how they have done so.

* 18. How do you think we can make the program better?

19. Is there anything else you would like to add about the tMHFA program or the research?

tMHFA 7-9 - Parent/Guardian and Teacher Feedback Survey

End of survey
